# The real-life effect of catechol-O-methyltransferase inhibition on non-motor symptoms in levodopa-treated Parkinson’s disease: opicapone versus entacapone

**DOI:** 10.1007/s00702-023-02603-y

**Published:** 2023-04-10

**Authors:** Valentina Leta, Daniel J. van Wamelen, Federico Aureli, Vinod Metta, Dhaval Trivedi, Pietro Cortelli, Carmen Rodriguez-Blazquez, Alexandra Rizos, K. Ray Chaudhuri

**Affiliations:** 1 Department of Basic and Clinical Neuroscience, King’s College London, Institute of Psychiatry, Psychology and Neuroscience, The Maurice Wohl Clinical Neuroscience Institute, Cutcombe Road, London, SE5 9RT UK; 2 Parkinson’s Foundation Center of Excellence, King’s College Hospital, London, UK; 3 Department of Neuroimaging, King’s College London, Institute of Psychiatry, Psychology and Neuroscience, London, UK; 4 Department of Neurology, Centre of Expertise for Parkinson and Movement Disorders, Radboud University Medical Centre, Donders Institute for Brain, Cognition and Behaviour, Nijmegen, The Netherlands; 5 Department of Biomedical and NeuroMotor Sciences (DIBINEM), Alma Mater Studiorum-University of Bologna, Bologna, Italy; 6 King’s College Hospital London, Dubai, United Arab Emirates; 7 National Centre of Epidemiology and CIBERNED, Institute of Health Carlos III, Madrid, Spain

**Keywords:** Parkinson’s disease, Non-motor symptoms, Sleep, Opicapone, Entacapone, Catechol-O-methyltransferase

## Abstract

**Objective:**

To evaluate the long-term, real-life effects on non-motor symptoms (NMS) of opicapone compared to entacapone in levodopa-treated people with Parkinson’s disease (PwP).

**Methods:**

A retrospective data analysis, with pre- and post-opicapone initiation data of 17 PwP with motor fluctuations compared to a comparable group of 18 PwP introduced on entacapone. The primary outcome was changes in the NMS Scale (NMSS) total score after 1-year follow-up. Secondary outcomes included changes in the NMSS domains, and Parkinson’s Disease Sleep Scale (PDSS) total and item scores after the same time span.

**Results:**

Groups were comparable for baseline demographics and Parkinson’s-related features (*p ≥ *0.314) as well as duration of follow-up (1.33 ± 0.66 years for PwP on opicapone and 1.23 ± 0.49 years for those on entacapone; *p = *0.858). PwP who were introduced on opicapone showed no changes in NMSS and PDSS total scores after 1 year (*p = *0.605 and *p = *0.507, respectively), whereas PwP who were introduced on entacapone showed significant worsening of NMSS and PDSS total scores at follow-up (*p = *0.005 and *p = *0.001, respectively). In neither group changes in individual NMSS domains from baseline to follow-up were observed (*p ≥ *0.288 for entacapone and *p ≥ *0.816 for opicapone, respectively). In PwP on entacapone significant worsening was seen in the distressing dreams, hallucinations, and limb numbness items of the PDSS (*p ≤ *0.05).

**Conclusions:**

Introduction of opicapone in real-life PwP with motor fluctuations seems to stabilise NMS burden and aspects of sleep dysfunction, in contrast to entacapone where there was a worsening of NMS burden and PDSS scores over 1 year follow-up.

## Introduction

Opicapone is a long-acting, third generation selective catechol‐o‐methyl transferase (COMT) inhibitor, which became available in Europe in 2016 and is indicated as adjunctive treatment to levodopa in people with Parkinson's disease (PwP) who experience motor fluctuations (Scott 2021; Leta et al. 2020). Significant beneficial effect of opicapone in reducing duration of motor ‘off’ episodes has been recently shown in the BIPARK I and BIPARK II studies, with the latter showing also a positive signal for some non-motor aspects including the non-motor symptoms (NMS) scale (NMSS) sleep/fatigue domain (Hauser et al. 2020; Lees et al. 2017; Oliveira et al. 2015). Similarly, the open-label single-arm OptiPark study showed a significant improvement in NMS burden at three months, largely driven by improvements in sleep/fatigue, mood/anxiety and miscellaneous domains of the NMS scale (Schofield et al. 2022; Reichmann et al. 2020). Other open label studies have suggested that both entacapone and tolcapone may also improve aspects of sleep dysfunction and NMS burden in levodopa-treated fluctuating PwP (Park et al. 2020; Müller 2014). However, direct real-life comparisons of these COMT inhibitors and their long-term effect on NMS and sleep dysfunction using validated scales and questionnaires are lacking. In this retrospective data analysis, we aimed to investigate the real-life, long-term effects of opicapone on NMS and sleep dysfunction in PwP with motor fluctuations compared to those who were introduced on entacapone.

## Materials and methods

In this retrospective data analysis, pre- and post-opicapone as well as pre- and post- entacapone initiation data were collected from the Non-Motor International Longitudinal Study (NILS) database. NILS is an observational longitudinal study addressing the range and natural history of NMS in PwP (https://www.gsttbrc.com/NILS) and has been running since 2011 (van Wamelen et al. 2021b). It involves 34 centres worldwide and has been adopted as a national study by the National Institute of Health Research in the United Kingdom and received relevant ethical approval (NRES SouthEast London REC3, 10084, 10/H0808/141). All participants gave written consent prior to study procedures in accordance with the Declaration of Helsinki.

For this analysis, only data collected at the Parkinson’s Foundation Centre of Excellence at King’s College Hospital (London, UK) were included. The primary outcome was pre- and post-opicapone initiation changes in the NMSS total score, compared to pre- and post-entacapone initiation changes after 1 year. The NMSS assesses NMS by severity (0–3) and frequency (1–4) and groups symptoms into nine domains as well as providing a total score for NMS (van Wamelen et al. 2021a). Secondary outcomes included changes in the NMSS domains, Parkinson’s Disease Sleep Scale (PDSS) total and individual 15 items scores of this scale at follow-up. The PDSS scores 15 items related to sleep dysfunction in PwP on a scale of 1 to 10 with lower scores indicated worse quality of sleep (Chaudhuri et al. 2002).

### Data analysis

Data were summarised descriptively, between-group differences at baseline tested using the Mann–Whitney U test or Chi Square test, where relevant, and changes in outcomes tested using the Wilcoxon signed-rank test. A *p* value of < 0.05 was considered statistically significant and Benjamini–Hochberg procedure was used to correct for multiple testing, where relevant. Statistical analyses were performed using the Statistical Package for the Social Sciences (SPSS), version 28.0 (IBM Corp., Armonk, NY, USA).

## Results

A total of 17 and 18 PwP, who were initiated on opicapone and entacapone, respectively, were identified. Baseline demographics and Parkinson’s disease-related data of participants is summarised in Table 1. Both groups were well-matched at baseline (*p ≥ *0.314; Table 1), including duration of follow-up (1.33 ± 0.66 years for PwP introduced on opicapone and 1.23 ± 0.49 years for those on entacapone; *p = *0.858).

**Table 1:** Baseline demographics and Parkinson’s disease-related data

	Entacapone (*N = *18)	Opicapone (*N = *17)	*p*
Age at assessment (years)	62.69 ± 11.81	55.94 ± 8.33	0.314
Sex (M/F)	13/5	14/3	0.560
H&Y	3 (2–3)	3 (2–3)	0.417
Disease duration (years)	7.25 ± 5.00	7.53 ± 3.99	0.695
LEDD (mg)	952.06 ± 374.69	780.74 ± 437.01	0.314
SCOPA motor total score	18.89 ± 9.77	15.41 ± 9.40	0.541
SCOPA motor	8.78 ± 5.55	6.12 ± 3.76	0.326
SCOPA ADL	7.78 ± 4.36	5.65 ± 4.14	0.416
SCOPA complications	2.33 ± 2.35	3.65 ± 2.69	0.416
NMSS total score	52.94 ± 34.79	43.88 ± 38.53	0.416
PDSS	104.11 ± 35.18	94.71 ± 41.58	0.416

The two groups are matched for demographics and Parkinson’s’ related features. Data presented as mean ± standard deviation or median (25th–75th percentile) or number. Group differences tested through Mann–Whitney *U* test or Chi Square test, where relevant, and corrected for multiple testing using Benjamini–Hochberg procedure

p: *p* value; LEDD: levodopa daily equivalent dose; SCOPA: Scales for Outcomes in Parkinson’s Disease; ADL: activities of daily living; NMSS: Non-Motor Symptoms Scale; PDSS: Parkinson’s Disease Sleep Scale; H&Y: Hoehn and Yahr

**Fig. 1:**
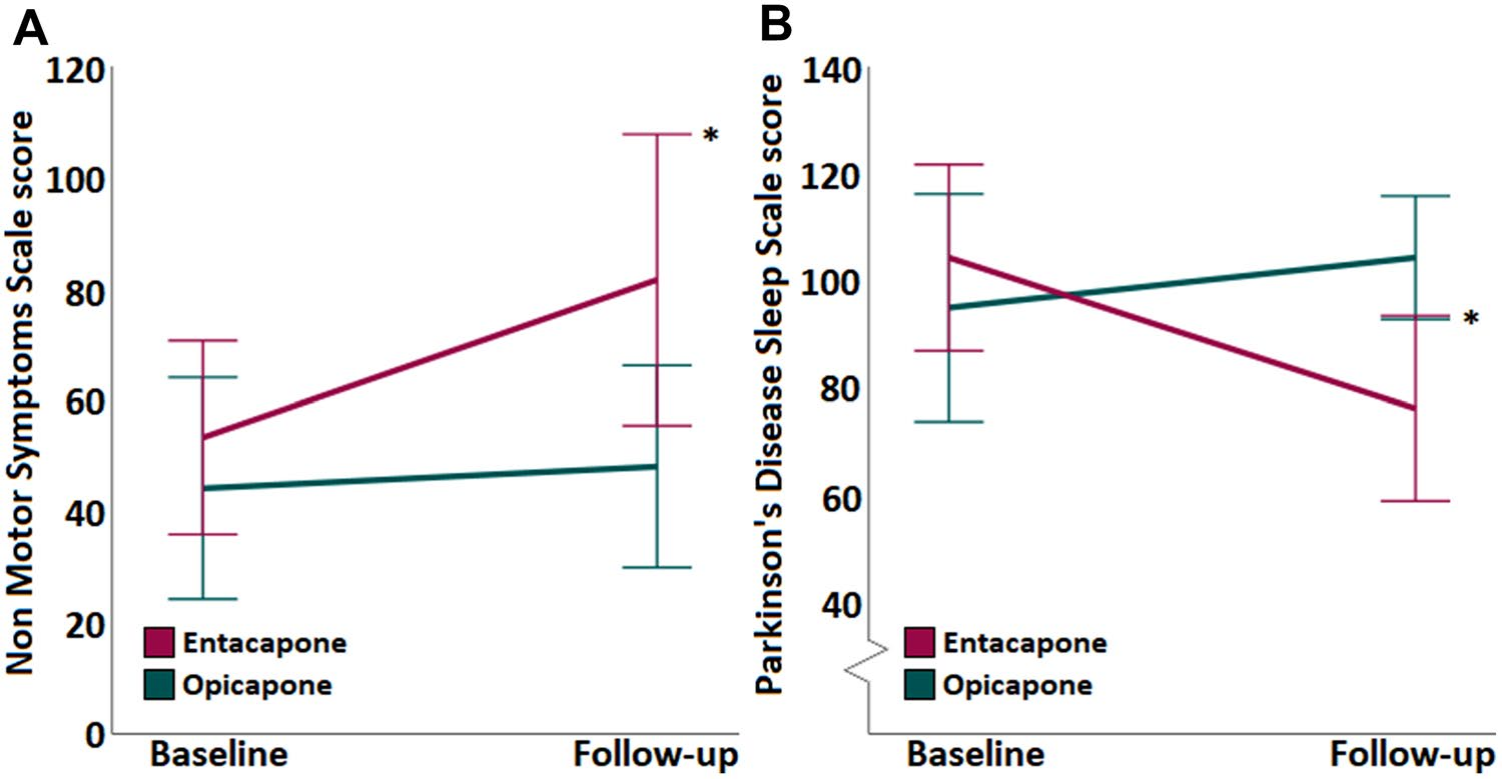
Non-motor (**A**) and sleep dysfunction (**B**) scores from baseline to follow-up following the introduction of a catechol-O-methyltransferase inhibitor in people with Parkinson’s disease. Please note that for the Parkinson’s disease sleep scale the lower the score, the more severe the sleep dysfunction. Duration of follow-up was 1.33 ± 0.66 years for the opicapone group and 1.23 ± 0.49 years for the entacapone group (*p = *0.858). * indicates statistically significant worsening of scores in the entacapone group (*p < *0.05)

PwP who were introduced on opicapone showed unchanged NMSS and PDSS total scores (*p = *0.605 and *p = *0.507, respectively; Fig. 1; Table 2), whereas PwP who were introduced on entacapone showed significantly worse NMSS and PDSS total scores at follow-up (*p = *0.005 and *p = *0.001, respectively; Fig. 1; Table 2). In neither group changes in individual NMSS domains from baseline to follow-up were observed (*p ≥ *0.288 for entacapone and *p ≥ *0.816 for opicapone, respectively). On the other hand, in PwP on entacapone significant worsening was seen in the distressing dreams, hallucinations, and limb numbness items scores of the PDSS (*p = *0.005) (Table 3).

**Table 2:** Non-motor outcomes from baseline to follow- up following the introduction of a catechol-O-methyltransferase inhibitor in people with Parkinson’s disease with motor fluctuations

	Entacapone (*N = *18)			Opicapone (*N = *17)		
	BL	FU	*p*	BL	FU	*p*
Primary outcome						
NMSS	52.95 ± 34.79	81.11 ± 52.38	**0.005**	43.88 ± 38.53	47.76 ± 35.09	0.605
Secondary outcome						
PDSS	104.11 ± 35.18	75.83 ± 34.94	**0.001**	94.71 ± 41.58	104.12 ± 22.53	0.507

While no significant changes were observed for non-motor scores in patients with Parkinson’s disease initiated on opicapone, worsening of non-motor scores were observed in patients with Parkinson’s disease initiated on entacapone at follow up. Please note that for the Parkinson’s disease sleep scale the lower the score, the more severe the sleep dysfunction. Data presented as mean ± standard deviation. Changes tested through Wilcoxon signed ranks test and statistically significant values highlighted in bold (*p* ≤ 0.05)

BL: baseline; FU: follow up; NMSS: Non-Motor Symptoms Scale; p:* p* value; PDSS: Parkinson’s Disease Sleep Scale

**Table 3:** Parkinson’s Disease Sleep Scale scores from baseline to follow-up following the introduction of a catechol-O-methyltransferase inhibitor in people with Parkinson’s disease with motor fluctuations

PDSS items	Entacapone (*N = *18)				Opicapone (*N = *17)			
	BL	FU	*p*	p*	BL	FU	*p*	p*
Overall sleep quality	4.67 ± 3.13	4.06 ± 2.28	0.590	0.590	5.80 ± 2.96	5.94 ± 2.24	0.887	0.959
Difficulties falling asleep	5.94 ± 4.11	4.29 ± 3.39	0.162	0.220	7.73 ± 2.28	7.25 ± 2.62	0.416	0.959
Difficulties staying asleep	5.50 ± 4.09	3.76 ± 3.51	0.092	0.178	4.67 ± 2.97	5.76 ± 2.56	0.063	0.473
Restlessness	7.33 ± 3.77	6.82 ± 3.21	0.322	0.374	7.20 ± 3.76	7.82 ± 3.36	0.719	0.959
Fidgeting	6.33 ± 4.10	5.18 ± 3.91	0.136	0.212	7.07 ± 3.41	7.82 ± 2.83	0.646	0.959
Distressing dreams	8.94 ± 1.96	5.59 ± 3.54	**0.002**	**0.030**	4.00 ± 2.20	8.29 ± 2.52	0.944	0.959
Distressing hallucinations	8.89 ± 1.88	6.65 ± 2.94	**0.008**	**0.050**	9.20 ± 1.37	9.53 ± 0.80	0.516	0.959
Nocturia	4.56 ± 4.32	2.84 ± 3.23	0.095	0.178	5.00 ± 3.85	4.93 ± 3.27	0.959	0.959
Incontinence	8.89 ± 2.06	7.41 ± 3.02	**0.035**	0.105	9.00 ± 1.53	8.65 ± 2.45	0.931	0.959
Numbness	8.06 ± 3.26	5.65 ± 3.45	**0.010**	**0.050**	6.93 ± 2.84	7.84 ± 2.22	0.043	0.473
Painful cramps	7.39 ± 3.26	6.12 ± 3.50	0.141	0.212	7.53 ± 2.97	6.88 ± 2.75	0.178	0.540
Early morning painful posturing	7.00 ± 3.94	6.00 ± 3.30	0.397	0.425	7.07 ± 3.63	7.76 ± 2.56	0.824	0.959
Tremor on waking	7.50 ± 3.76	6.12 ± 3.57	0.085	0.178	8.73 ± 1.58	8.29 ± 3.20	0.167	0.540
Tiredness after waking	5.72 ± 3.80	4.29 ± 2.52	0.324	0.374	7.47 ± 2.95	4.38 ± 2.39	0.180	0.540
Daytime sleepiness	7.39 ± 3.36	5.41 ± 3.91	**0.016**	0.060	7.53 ± 2.42	7.24 ± 3.17	0.893	0.959

Data presented as mean ± standard deviation. Changes tested through Wilcoxon signed ranks test. Please note that for the Parkinson’s disease sleep scale the lower the score, the more severe the sleep dysfunction

PDSS: Parkinson’s Disease Sleep Scale; p*: *p* value corrected for multiple testing using Benjamini–Hochberg procedure

Statistically significant values highlighted in bold (*p* ≤ 0.05)

## Discussion

In this retrospective data analysis, we observed that PwP with motor fluctuations introduced on opicapone showed unchanged NMSS and PDSS scores after 1-year follow-up, whereas both scales showed significant worsening in PwP introduced on entacapone over the same time span. To the best of our knowledge, this is the first data analysis comparing 1-year non-motor effects of opicapone with entacapone in PwP. Despite the limitations of a retrospective data analysis, we provide further real-life evidence for non-motor benefits of opicapone on NMS in PwP which forms the basis of international clinical trials, such as OASIS (ClinicalTrials.gov Identifier: NCT04986995) and OCEAN (ClinicalTrials.gov Identifier: NCT04986982) (Chaudhuri et al. 2022).

While the focus of trials on COMT inhibitors, including opicapone, has been on the motor effects with specific attention for the improvement of motor fluctuations (Schofield et al. 2022; Reichmann et al. 2020; Scott 2021; Hauser et al. 2020; Lees et al. 2017), less evidence on their beneficial effect on NMS is available at present. Some studies have previously shown that opicapone improves NMS, such as the OptiPark and the OPEN-PD study; however, these studies often had limited follow-up (up to 6 months) and a comparator group was lacking (Schofield et al. 2022; Reichmann et al. 2020; Santos García et al. 2022). Our data indicates that NMSS and PDSS scores remain unchanged in the opicapone group in a real-life cohort of levodopa-treated fluctuating PwP after 1 year although we were not informed about possible initial improvements following the introduction of opicapone as already been shown in previously published studies (Schofield et al. 2022; Reichmann et al. 2020; Santos García et al. 2022). Nonetheless, our data suggests a sustained non-motor beneficial effect of opicapone and superiority of opicapone over entacapone in relation to these effects as in the entacapone arm a significant worsening of both NMSS and PDSS scores was observed in spite of similar mechanism of action of COMT inhibition. The prolonged action of opicapone once daily compared to multiple daily intakes of the short acting entacapone may explain the difference and a greater beneficial effect on aspects of non-motor fluctuations (NMF) with opicapone (Schofield et al. 2022). We were unable to measure the extent of NMF in these patients as the analysis was retrospective and tools to measure NMF in the clinic, such as the NMF subscale of Movement Disorder Society Non-Motor Rating Scale (MDS-NMS), have only recently become available (Chaudhuri et al. 2020). We also speculate that the longer activity of opicapone compared to entacapone might explain the sustained beneficial effect on aspects of sleep dysfunction (restless legs-like symptoms and vivid dreams) which could be part of NMF and can be also underpinned by dopaminergic dysfunction (Chaudhuri and Schapira 2009).

As with any study, it is important to acknowledge the limitations to our analyses. As we used real-life retrospective data, the size of our patient population was relatively limited, and further limitations came in the form of the open label prescription of opicapone and entacapone in patients with motor fluctuations who were, therefore, not selected for NMS. Finally, also the lack of an assessment between baseline and year 1 could be perceived as a limitation as it did not allow us to determine whether the stabilisation of non-motor burden in PwP treated with opicapone was a return to baseline after a possible initial improvement. Nonetheless, we feel our findings are specifically relevant as this data does not suffer from bias that affects clinical trials where real-life patients are often excluded. Additional value comes from the presence of a well-matched comparator group on entacapone, where worsening likely reflected NMS worsening related to disease progression, the use of standardised non-motor and sleep assessments, and the relatively long follow-up period. Further studies on opicapone with a focus on NMS outcomes and cohorts selected for specific NMS are encouraged, which would, at some point in the future, allow the addition of NMS as a possible indication to initiate COMT inhibitors in PwP.

In summary, we observed that the initiation of opicapone in PwP with motor fluctuations seemed to stabilise non-motor burden after 1 year of follow-up, unlike in PwP on entacapone for which a significant worsening in NMS and sleep problems were observed. Despite the above limitations, this study provides further evidence for the use of opicapone in PwP and seems to confirm the usefulness of opicapone for the treatment of NMS and sleep dysfunction in the long term.

## Data Availability

Data supporting the findings of this analysis on the NILS database are available from the corresponding author upon reasonable request.
